# A Comparative Analysis on Prediction Performance of Regression Models during Machining of Composite Materials

**DOI:** 10.3390/ma14216689

**Published:** 2021-11-06

**Authors:** Shibaprasad Bhattacharya, Kanak Kalita, Robert Čep, Shankar Chakraborty

**Affiliations:** 1 Department of Production Engineering, Jadavpur University, Kolkata 700030, India; shibaprasadb@yahoo.com; 2 Department of Mechanical Engineering, Vel Tech Rangarajan Dr. Sagunthala R&D Institute of Science and Technology, Avadi 600062, India; 3 Department of Machining, Assembly and Engineering Metrology, Faculty of Mechanical Engineering, VŠB—Technical University of Ostrava, 17. Listopadu 2172/15, 708 00 Ostrava, Czech Republic; robert.cep@vsb.cz

**Keywords:** regression, model, turning, drilling, composite material

## Abstract

Modeling the interrelationships between the input parameters and outputs (responses) in any machining processes is essential to understand the process behavior and material removal mechanism. The developed models can also act as effective prediction tools in envisaging the tentative values of the responses for given sets of input parameters. In this paper, the application potentialities of nine different regression models, such as linear regression (LR), polynomial regression (PR), support vector regression (SVR), principal component regression (PCR), quantile regression, median regression, ridge regression, lasso regression and elastic net regression are explored in accurately predicting response values during turning and drilling operations of composite materials. Their prediction performance is also contrasted using four statistical metrics, i.e., mean absolute percentage error, root mean squared percentage error, root mean squared logarithmic error and root relative squared error. Based on the lower values of those metrics and Friedman rank and aligned rank tests, SVR emerges out as the best performing model, whereas the prediction performance of median regression is worst. The results of the Wilcoxon test based on the drilling dataset identify the existence of statistically significant differences between the performances of LR and PCR, and PR and median regression models.

## 1. Introduction

A composite material usually consisting of a combination of two or more materials with varying physical and chemical properties has superior characteristics as compared to its individual constituents. Without losing the properties of the entities, they are combined together, contributing to the most useful properties of a composite material for a special purpose application [1]. Several advantageous properties of composite materials, such as high impact strength, stiffness, corrosion resistance, strength-to-weight ratio, thermal conductivity, dimensional stability, customized surface finish, lightweight, etc., have made them a popular choice in manufacturing of aerospace structures, electrical equipment, pipes and tanks, laminated beams, etc. Thus, a composite material has multiple desirable properties which cannot be found in a single traditional material.

Among different types of composite materials, fiber-reinforced polymer (FRP) composites have a polymer matrix which is reinforced with an artificial or natural fiber (i.e., carbon, glass or aramid). In FRP composites, the matrix protects the fibers from environmental and external damage, while the fibers provide strength and stiffness resisting crack generation and failure of the base material. On the other hand, in metal matrix composites (MMCs), the matrix is usually made of a lighter metal (i.e., aluminum, magnesium, etc.) which is reinforced with silicon carbide or ceramics to impart higher strength and toughness with extremely low coefficient of thermal expansion. The MMCs are more suitable in many industrial applications requiring long-term exposure to severe environments than FRP composites. Due to their high yield strength and modulus of elasticity, MMCs can be plastically deformed and strengthened using various thermal and mechanical treatments. Due to reinforcement in the base material, these composites impose severe problems during their machining. Unlike plastic deformation in conventional metal cutting operation, there is no chip formation while machining FRP composites. In this case, material removal takes place due to shattering, which causes rupture of the embedded fibers due to the action of sharp cutting edge and also abrasion of the cutting edge causing rapid tool wear [2,3,4]. In case of machining of MMCs, the hard reinforcement particles when come in contact with the tool, start forming built-up edges. This leads to generation of rough machined surface and is also responsible for high tool wear [5,6].

It has been noticed that during machining of composite materials, various input parameters, such as cutting speed, feed rate, depth of cut, type of the tool material, tool nose radius, etc., in turning; spindle speed, feed rate, configuration of the drill bit, drill diameter, etc., during drilling; and cutting speed, depth of cut, feed rate, configuration of the milling cutter, etc., in milling significantly affect the process outputs, mainly in the form of material removal rate (MRR), surface roughness, tool wear rate, machining time, tool tip vibration, energy consumption, etc. Thus, to understand the process behavior and study the influences of the input parameters on the responses, development of a mathematical/statistical model is quite useful. It can also act as a prediction tool in envisaging the response values for given sets of input parameters and help in determining the optimal parametric intermix to achieve the target responses. In this direction, application of response surface methodology (RSM)-based meta-modeling has been quite popular among the researchers [7,8,9,10,11,12,13,14] due to its ability to derive higher order and interaction effects between the input parameters with a smaller number of experimental data. Being a local analysis, the surface developed by this technique is supposed to be invalid for regions other than the considered ranges of the input parameters. In RSM, it is also not correct to assume that all the systems with curvature are compatible with a second-order polynomial equation. Artificial neural networks have also evolved out as effective modeling tools to study the underlying relationships between the input parameters and responses during machining of composite materials [15,16,17]. However, they are black-box type of approaches, having hardware dependency, unexplained structure and functioning of the network, and difficulty in deriving the optimal network architecture. In an attempt to avoid the drawbacks of ANN, Sheelwant et al. [18] integrated it with genetic algorithm (GA) for optimization of the input parameters during processing of Al-TiB2 MMC. Abhishek et al. [19] compared the predictive performance of GA and adaptive neuro-fuzzy interference system (ANFIS) while drilling GFRP materials, and proved the superiority of ANFIS model in predicting thrust force and average surface roughness (Ra) values. Laghari et al. [20] applied an evolutionary algorithm in the form of particle swarm optimization (PSO) technique for prediction and optimization of SiCp/Al MMC machining process. An excellent review on the applications of different soft computing techniques (GA, RSM, ANN, Taguchi methodology, PSO and fuzzy logic) for prediction of the process behavior during turning, drilling, milling and grinding operations of MMCs can be available in [21].

In statistics, regression analysis consists of a set of processes for representing the relationships between a dependent variable and one or more independent variables. It is basically employed for two main purposes, i.e., prediction and forecasting in machine learning, and development of causal relationships between the independent and dependent variables in statistical analysis. There are varieties of regression models, such as linear regression (LR), polynomial regression (PR), support vector regression (SVR), principal component regression (PCR), quantile regression, median regression, ridge regression, lasso regression, elastic net regression, logistic regression, ordinal regression, Poisson regression, Cox regression, Tobit regression, etc.

ML applications, despite its tremendous strides in some other fields, is at a nascent stage in manufacturing/machining sciences. The primary goal of this work is to analyze the utility of various ML-based regression methods in predictive modeling of machining processes. In this paper, LR, PR, SVR, PCR, quantile regression, median regression, ridge regression, lasso regression and elastic net regression are considered because of their ability to deal with continuous data for predicting the response values during turning and drilling operations of composite materials based on two past experimental datasets. To the best of the authors’ knowledge, these regression models have been individually applied as prediction tools in separate machining processes, and no study has been conducted dealing with their applications in a single research framework. The predictive performance of the considered regression models is contrasted using four statistical error estimators, i.e., mean absolute percentage error (MAPE), root mean squared percentage error (RMSPE), root mean squared logarithmic error (RMSLE) and root relative squared error (RRSE) for both the case studies. Finally, two non-parametric tests in the form of the Friedman test and Wilcoxon test are performed to respectively identify the best performing regression model and statistically significant differences between those models.

## 2. Machine Learning-Based Predictive Modeling

### 2.1. Linear Regression

It is the simplest form of the regression models where the relationship between independent and dependent variables is considered to be linear. It only takes into account the main effects of the independent variables on the dependent variable, having the following form:

(1) $$y=\beta_{0}+\beta_{1}x_{1}+\cdots +\beta_{i}x_{i}+\cdots +\beta_{n}x_{n}+\varepsilon$$

where y is the dependent variable, $\beta_{0}$ is the intercept, $\beta_{i}$ is the coefficient of ith independent variable, $x_{i}$ is the *i*th independent variable (*i* = 1,2,…, n) and ε is the error term. Thus, based on simple linear equation, values of the responses for any combination of the input parameters within the specified range can be predicted.

### 2.2. Polynomial Regression

Unlike multivariate LR, PR model is usually developed while considering higher-order terms of the input parameters (independent variables). Both LR and PR models determine the corresponding coefficient values based on ordinary least squares estimator. In this paper, PR models of order two are developed which can be expressed as below:

(2) $$y=\beta_{0}+\beta_{1}x_{1}+\cdots +\beta_{i}x_{i}+\cdots +\beta_{n}x_{n}+\beta_{11}x_{1}{}^{2}+\cdots +\beta_{ii}x_{i}{}^{2}+\cdots +\beta_{nn}x_{n}{}^{2}+\varepsilon$$

where $\beta_{ii}$ is the coefficient of $x_{i}{}^{2}$ term.

### 2.3. Support Vector Regression

The SVR is a supervised learning technique, applied both for classification and regression, and is based on the principle of support vector machine (SVM), which develops a hyperplane between two sets of data [22,23]. A margin is created while developing two parallel hyperplanes, each on the opposite side, and its width reaches to the maximum at optimal solution. The optimal separation (solution) is achieved at minimum generalization error of the model, thus ensuring highest margin between the two hyperplanes. The data subset representing the optimal margin is known as support vector.

In SVM, dimension of the classified vectors has less influence on its performance unlike other conventional regression models. It employs a set of training data to learn and develop a model in order to minimize the generalization error when its performance is validated with different sets of testing data. Although it is mainly applied for solving classification problems, but after the introduction of SVR, it has received a great interest among the research community in solving regression problems which are quite difficult to solve by the conventional models. As it has very few tuning parameters, the corresponding computational effort greatly reduces while searching out its appropriate architecture for a given problem. Having the ability to solve both linear and non-linear models, it basically employs non-linear kernel functions (such as polynomial) to derive the optimal solutions for non-linear models.

### 2.4. Principal Component Regression

The PCR model combines both principal component analysis (PCA) and least squares regression [24]. Its application starts with developing a stepwise regression with a dependent variable y and a set of independent variables x for deriving *p* statistically significant independent variables (less than 0.05) and revealing the presence of multicollinearity among the p independent variables. A PCA is then performed with p independent variables for transforming a set of correlated variables to a set of uncorrelated principal components while indicating information quantities of different sets of principal components. In the subsequent steps, values of standardized dependent variable, p standardized independent variables and p principal components are determined for developing p standardized PCR models [25]. The standardized PCR model is thus formulated with the first principal component and the other principal components are added backwards one by one to derive p standardized PCR models. In this paper, all the input parameters for the considered turning and drilling processes are treated as the principal components.

### 2.5. Quantile Regression

Quantile regression is a technique to estimate relationship between a set of variables for all portions of a given probability distribution. While the conventional regression models provide information with respect to mean values of the distributions for a set of regressors, it computes several different regression models for various percentage points of the distribution while providing a complete depiction of the data [26]. For Tth quantile, the area under the probability distribution curve can be split into two sections, i.e., one with area below the *T*th quantile and the other with area (1−T) above it. Thus, the regression model for *T*th quantile can be represented as below:

(3) $$y=\beta_{0}+\beta_{1}{}^{T}x_{1}{}^{T}+\beta_{2}{}^{T}x_{2}{}^{T}+\ldots +\beta_{n}{}^{T}x_{n}{}^{T}+\varepsilon^{T}$$

In multivariate regression models, change in the conditional mean of the dependent variable related to a change in the regressor (independent variables) is specified, while quantile regression specifies changes in the conditional quantile. Thus, it can be considered as an extension of multivariate regression models. This model helps in inspecting the rate of change of the dependent variable by quantiles. When the model is developed for 50th quantile, it is called median regression.

### 2.6. Median Regression

It is already stated that the 50th quantile regression is known as median regression. Median regression is also sometimes referred to as LAV (least absolute-value) regression as its parameters are estimated by minimizing the sum of absolute value of the residuals. If covariates are absent in the median regression model, the calculated intercept would be the usual estimate of the median [27]. The adjusted median computed using LAV is relatively insensitive to outliers as compared to LR models. The following equation for median regression can now be derived from quantile regression:

(4) $$y=\beta_{0}+\beta_{1}{}^{0.5}x_{1}{}^{0.5}+\beta_{2}{}^{0.5}x_{2}{}^{0.5}+\ldots +\beta_{n}{}^{0.5}x_{n}{}^{0.5}+\varepsilon^{0.5}$$

### 2.7. Ridge Regression

As multivariate LR models are based on least squares estimates, they do not perform well for ill-conditioned data with respect to both prediction accuracy and model size. While deriving the optimal fit to the estimation data, least squares often do not perform well for new data (outside the region of the estimation data). To overcome these drawbacks of ordinary least squares estimates, several regularized regression models, such as ridge regression have evolved out since the last few decades.

In ridge regression, the main focus is to determine an appropriate smaller value of k to provide the least squares estimates without any prior information [28]. A ridge analysis is based on the original data or principal components. The orthogonality of both the data and priors provides estimates which are simple weighted averages of the likelihood estimate and the prior mean. These estimates with the largest variances are maximally shrunk, and larger values of k force all these estimates closer to zero. It does not reduce the coefficients to absolute zero and thus, cannot eliminate the statistically insignificant predictors.

### 2.8. Lasso Regression

The conventional multivariate regression models usually suffer from the problems of overfitting of data and overestimation (how well the model would perform to explain the observed variability using the considered variables). Overfitting occurs due to presence of statistically insignificant terms in the model, that inflates the training goodness-of-fit. They tend to perform poorly while predicting dependent variables having extremum risk. The least absolute shrinkage and selection operator regression i.e., lasso can effectively address both the problems. It is a shrinkage and variable selection method for developing regression models. It primarily aims to identify variables and corresponding coefficients to develop a model with minimum prediction error [29]. This can be attained while imposing a constraint on the model parameters to shrink the regression coefficients towards zero, i.e., by forcing sum of absolute values of the coefficients to be less than a fixed threshold (λ). After shrinkage, variables having regression coefficients of zero are excluded from the model. In this technique, λ is determined based on an automated k-fold cross-validation. k equi-sized sub-samples are generated from the initial dataset. (k−1) sub-samples are employed for developing the corresponding regression model. The remaining sub-sample is utilized for model validation. This procedure is repeated for k number of times, with each one of the k sub-samples being used for validation and the others for model development. The k separate validation results for a range of λ values and the most preferred value of λ are combined together to formulate the final model. Its main advantage is that it minimizes overfitting of data and may outperform other regression models for a particular set of tuning parameters.

### 2.9. Elastic Net Regression

Elastic net is an amalgam of lasso and ridge regression models, combining both the principles of shrinkage and variable selection [30]. It is extremely suitable for analyzing high-dimensional data which is quite robust against extreme correlations among the predictor variables. The lasso part of elastic net helps in automatic variable selection, whereas, ridge part aids in group selection while stabilizing the solution paths in regard to random sampling, which improves the prediction accuracy. With the help of grouping effect during variable selection, a group of highly correlated variables tends to have coefficients of similar magnitude. It can select groups of correlated features when the groups are not known in advance. For developing the corresponding model, elastic net adopts a combined penalty of lasso and ridge regression penalties. The penalty parameter α determines the weight to be provided to lasso or ridge regression. The elastic net with α as 0 is equivalent to ridge regression. On the other hand, the elastic net with α close to 1 behaves much like a lasso, while removing any degeneracy and odd behavior due to high correlations among the predictor variables. It has been noticed that the application of elastic net can result in lower mean squared errors for correlated variables.

It has already been mentioned that this paper focuses on the applications of nine different regression models as prediction tools during turning and drilling operations of composite materials. To have better performance of some of these models, values of the corresponding tuning parameters are chosen based on 5-fold cross-validation, as shown in Table 1 for both the machining processes. The value of λ adds a penalty in a given regression model. With its higher values, flexibility of the regression fit decreases, leading to lower variance but increased bias. In elastic net, value of α helps to reach a trade-off between ridge and lasso regression models. It behaves like ridge for α=0, and α=1 corresponds to lasso.

### 2.10. Statistical Metrics

In this paper, to validate the prediction performance of the nine regression models, four statistical error estimators, i.e., MAPE, RMSPE, RMSLE and RRSE are considered [31]. The MAPE compares the actual ($A_{i}$) and the predicted ($P_{i}$) responses in terms of percentage error. The RMSPE is a well-accepted measure to appraise goodness-of-fit of a regression model to best describe the average percent error during prediction of the response values. RMSLE, use of logarithm helps in estimating the percentual variation between the $A_{i}$ and $P_{i}$ response values. In this measure, small differences between small $A_{i}$ and $P_{i}$ response values are treated similarly as big differences between $A_{i}$ and $P_{i}$ response values. The RRSE is calculated by first finding the total squared error and then normalizing it by dividing with the total squared error of the simple predictor. The MAPE, RMSPE, RMSLE and RRSE are computed as:

(5) $$\mathrm{MAPE}=\frac{1}{n}\sum_{i=1}^{n}\left|\frac{A_{i}\;-\;P_{i}}{A_{i}}\right|\times 100$$

(6) $$\mathrm{RMSPE}=\sqrt{\frac{1}{n}\sum_{i=1}^{n}{\left(\frac{A_{i}\;-P_{i}}{A_{i}}\right)}^{2}}\times 100$$

(7) $$\mathrm{RMSLE}=\sqrt{\frac{1}{n}\sum_{i=1}^{n}{\left(\log(P_{i}+1)\;-\;\log(A_{i}\;+\;1)\right)}^{2}}$$

(8) $$\mathrm{RRSE}=\sqrt{\frac{\sum_{i=1}^{n}{\left(P_{i}\;-\;A_{i}\right)}^{2}}{\sum_{i=1}^{n}{\left(A_{i}\;-\;\bar{A}\right)}^{2}}}$$

where $A_{i}$ and $P_{i}$ are respectively the values of actual and predicted responses, $\bar{A}$ and $\bar{P}$ are the averages of all the actual and predicted responses respectively, and n is the number of test data.

## 3. Prediction of Responses during Machining of Composite Materials

### 3.1. Turning

Using a CNC lathe and based on Taguchi’s L_16_ orthogonal array as the experimental design plan, Laghari et al. [20] conducted 16 experiments on SiCp/Al MMC with cutting speed (*v_c_*) (in m/min), feed rate (*f*) (in mm/rev) and depth of cut (*a_p_*) (in mm) as the turning parameters, and Ra (in μm) and tool life (TL) (in min) as the process outputs (responses). Turning operations were performed on the considered work material using a carbide cutting tool under dry machining conditions. Each of the turning parameters was varied at four different operating levels to study their effects on the responses. The measured response values at varying combinations of the turning parameters are provided in Table 2. Among these 16 experimental observations, 11 datasets are randomly selected for training the considered regression models, whereas the remaining five are adopted for testing purposes.

Now, for this turning process, to explore the applicability and potentiality of the considered regression models, and validate their prediction performance, the corresponding regression models are developed using the open-source programming language R (version 4.0.5). The related LR and PR-based models for Ra and TL are provided as below:

For Ra:
  LR: Ra = 1.36 − 0.040 × *v_c_* − 8.015 × *f* − 0.244 × *a_p_* (9)
  PR: Ra = 1.735 − 0.1248 × *v_c_* + 26.02 × *f* − 0.6385 × *a_p_* + 0.00269 × *v_c_*^2^ − 0.09725 × *f*^2^ + 0.2303 × *a_p_*^2^ (10)
For TL:
  LR: TL = 11.2045 − 0.274 × *v_c_* − 145.13 × *f* − 0.6581 × *a_p_* (11)
  PR: TL = 20.65 − 0.6824 × *v_c_* + 914.3 × *f* − 3.622 × *a_p_* − 0.013 × *v_c_*^2^ − 21980 × *f*^2^ + 1.7.33 × *a_p_*^2^ (12)

**Figure 1:**
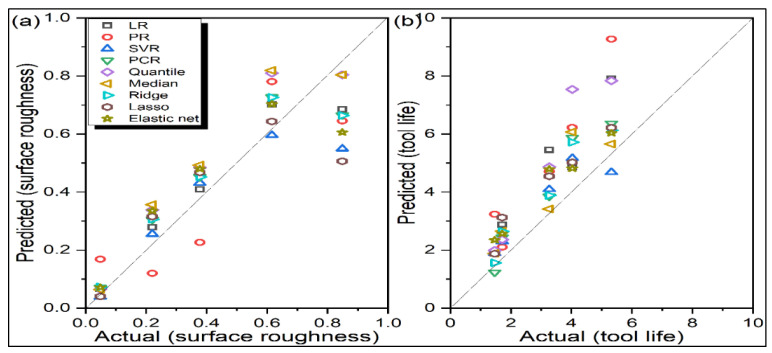
Actual versus predicted responses for turning (**a**) Ra (**b**) TL.

**Figure 2:**
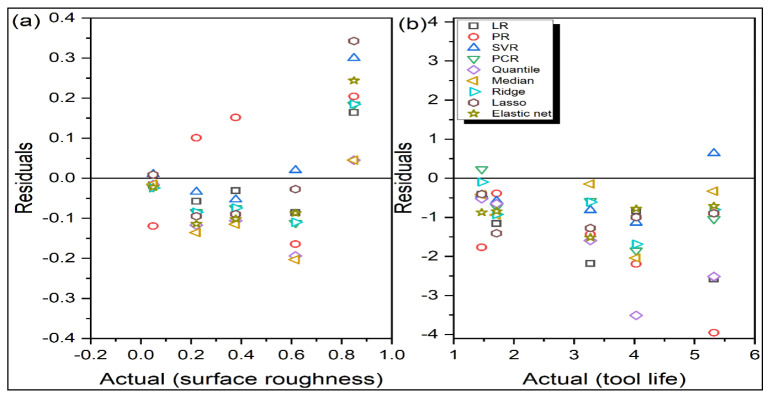
Actual responses versus residuals for turning (**a**) Ra (**b**) TL.

**Figure 3:**
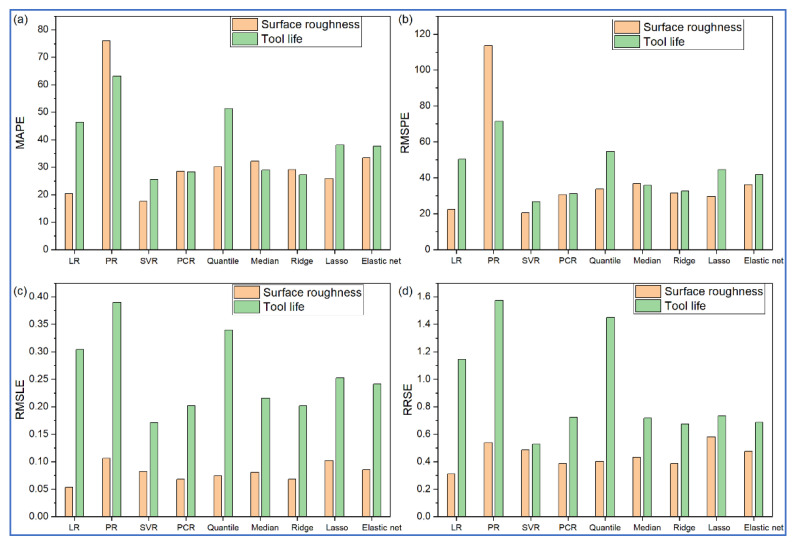
Prediction performance of various regression models for turning. Prediction errors in terms of (**a**) MAPE (**b**) RMSPE (**c**) RMSLE (**d**) RRSE.

Table 3 and Table 4, respectively, show Ra and TL’s predicted values during turning operation for all the nine regression models. On the other hand, Figure 1 depicts the actual versus predicted responses for the testing data by the considered regression models. The closer the test data points are to the diagonal identity line, the better is the prediction performance with lesser error. If there is an overlap of a data point on the identity line, it indicates 100% prediction accuracy for that data point. Similarly in Figure 2, if the data points lie on the zero line, there would be no residue (error) after prediction. The larger the vertical distance of a data point from the zero line, the larger is the residue. Positive residues indicate underprediction, whereas negative residues denote overprediction by the corresponding regression model. Conversely, for Figure 1, values above the identity line indicate over-prediction, and below the identity line, the regression model indicates underprediction. Thus, from Figure 1a and Figure 2a, it is observed that PR has large residues for all the test data points. On the other hand, the predictions are quite accurate for the SVR model baring one test data point. Small residues are also noticed for LR models. For tool life, all the regression models are found to be overpredicting, as revealed from Figure 1b and Figure 2b. Here too, PR-based predictions have high residues. However, having simple mathematical formulation and structure, LR seems to be the most adequate model in correctly predicting both responses. Values of all the statistical error estimators, i.e., MAPE, RMSPE, RMSLE and RRSE, are now plotted in Figure 3. This figure reveals that SVR has the minimum values for all the error metrics, whereas, PR has high prediction errors.

In an attempt to identify the best and worst-performing regression models, and statistically significant differences between pairs of the regression models based on the predicted response values, Friedman test and Wilcoxon test are respectively carried out [32]. The Friedman ranks and aligned ranks are respectively provided in Table 5 and Table 6 for Ra values during turning operation of SiCp/Al MMC. While assigning aligned ranks using the Friedman test, the average prediction performance by all the models is first computed for each test dataset. The differences between the performances of all the models and the average are then calculated, and are subsequently ranked. The results of both Friedman rank and aligned rank tests identify SVR as the best performing regression model (having the minimum average ranks) for the considered test dataset, where the prediction performance of median regression is not at all satisfactory. The results of Wilcoxon test for Ra, as exhibited in Table 7, exhibit no statistically significant difference between any pair of the regression models with respect to their prediction performance. Similar observations are also noticed for TL response during the said turning operation.

### 3.2. Drilling

Chaudhary et al. [33] performed drilling operation on aluminum MMCs with spindle speed (*S*) (in rpm), feed rate (*f*) (in mm/rev) and point angle (*P*) (in degree) as the input parameters, and MRR (in mm^3^/min), Ra (in μm) and oversize (OS) (in mm) as the responses. Based on a central composite design plan, 20 experiments were conducted while varying the drilling parameters at three different levels. Table 8 shows the values of different drilling parameters and responses at various experimental trials. Among 20 experimental runs, 16 trials are chosen for training of the regression models and their prediction performance is evaluated using the remaining six observations.

Like the turning process, the corresponding regression models are now developed for this drilling process using the considered nine modeling techniques. The following are the LR and PR-based regression models for MRR, Ra and OS during the drilling operation of aluminum MMCs.

For MRR:
  LR: MRR = −542.3 + 0.4817 × *S* + 379.4433 × *f* + 4.41315 × *P* (13)
  PR: MRR = 1246 − 2.047 × *S* + 567.3 × *f* − 28.39 × *P* − 0.00413 × *S*^2^ − 0.06041 × *f*^2^ + 0.1391 × *P*^2^ (14)
For Ra:
  LR: Ra = 10.959 − 0.002426 × *S* − 2.1204 × *f* − 0.006932 × *P* (15)
  PR: Ra = 636.1 + 0.04803 × *S* − 0.2518 × *f* − 11.03 × *P* − 0.000129 × *S*^2^ − 71.61 × *f*^2^ + 0.0478 × *P*^2^ (16)
For OS:
  LR: OS = 1021 − 0.00002 × *S* − 0.0959 × *f* − 0.008122 × *P* (17)
  PR: OS = 30.45 − 0.000416 × *S* + 0.6571 × *f* − 0.5233 × *P* − 0.000001 × *S*^2^ − 2.321 × *f*^2^ + 2.244 × *P*^2^ (18)

**Figure 4:**
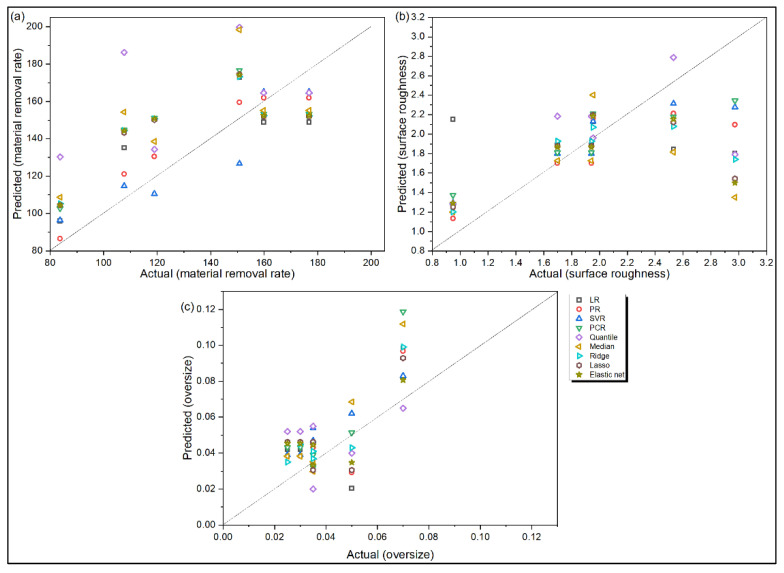
Actual versus predicted responses for drilling (**a**) MRR (**b**) Ra and (**c**) OS.

**Figure 5:**
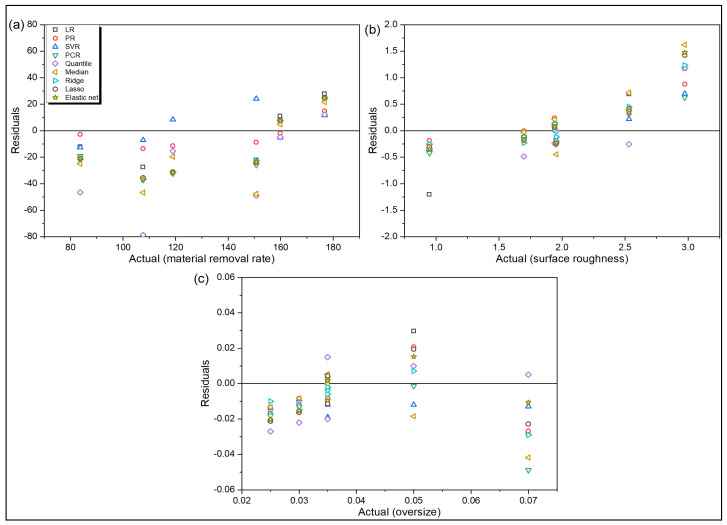
Actual responses versus residuals for drilling (**a**) MRR (**b**) Ra (**c**) OS.

**Figure 6:**
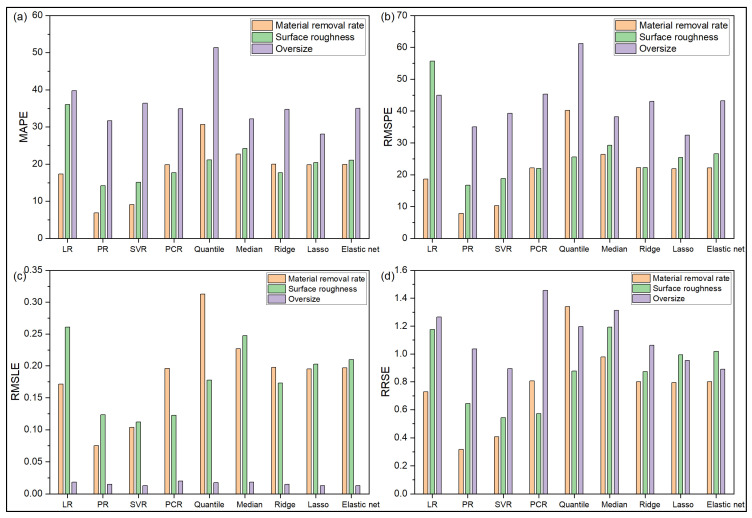
Predictive performance of various regression models for drilling operation. Prediction errors in terms of (**a**) MAPE (**b**) RMSPE (**c**) RMSLE (**d**) RRSE.

Figure 4a and Figure 5a depict that LR, PR and SVR are the top three models for accurate prediction of MRR values in the said drilling operation. Quantile and median regression models have the largest residues. Furthermore, the overprediction errors for all the models are observed to be larger than their corresponding underprediction errors. On the contrary, during prediction of Ra value, the order of magnitude of error for underprediction is larger than that for overprediction, as revealed from Figure 4b and Figure 5b. Here, SVR is observed to the best performing model, followed by PR and PCR. From Figure 4c and Figure 5c, it can be unveiled that there are more overprediction errors than underprediction errors for OS response, and SVR appears to be the best performing regression model, followed by LR. When the values various statistical error estimators are plotted in Figure 6, it can be noticed that SVR has the superior prediction performance, followed by LR and PR models. On the other hand, ridge and median regression models have worse prediction performance. Like the turning problem, applications of Friedman rank and aligned rank tests (not shown here due to paucity of space) also recognize SVR as the best performing regression model for predicting all the response values for the said drilling process. Table 9 depicts the calculated *p*-values of Wilcoxon test for MRR, which reveal significant differences in the prediction performance between LR and PCR, and PR and quantile regression models. Similar differences are also noticed for Ra and OS responses for the said drilling process.

## 4. Conclusions

This paper deals with exploring the application potentiality of nine different types of regression models, i.e., LR, PR, SVR, PCR, quantile regression, median regression, ridge regression, lasso regression and elastic net regression as effective prediction tools for envisaging the response values during turning and drilling operations of composite materials. Two past experimental datasets are employed here for training and subsequent validation of the developed regression models. Values of the required model tuning parameters are evaluated using 5-fold cross-validation approach. It is noticed that for both the machining processes, SVR emerges out as the best regression model with minimum values of MAPE, RMSPE, RMSLE and RRSE, followed by LR and PR models. On the contrary, ridge and median regression models have poor prediction performance. Results of Friedman rank and aligned rank tests also portray the same observations. The superiority of SVR model for the two cases studies reported in the paper may be due to its smaller number of tuning parameters, robustness, and capability to deal with both linear and non-linear models. The application of another non-parametric test (Wilcoxon test) identifies differences in the prediction performances between LR and PCR, and PR and quantile regression models at 5% significance level for the drilling process. In this paper, prediction performance of all the nine regression models is contrasted using small experimental datasets. Better and more accurate results may be expected while applying these models for large datasets. As a future scope, other regression models dealing with categorical variables, such as logistic regression, Cox regression, Tobit regression, etc., may be employed as prediction tools in real-time machining environment.

## Figures and Tables

**Table 1:** Values of the corresponding tuning parameters.

Model	Parameter	Turning		Drilling		
		Ra	TL	MRR	Ra	OS
Ridge	*λ*	0.001995	0.316228	5.01	1	0.005012
Lasso	*λ*	0.1	0.501187	2.51	0.125893	0.00631
Elastic net	*λ*	0.041822	0.808134	3.436782	0.187975	0.005182
	*α*	0.803634	0.897613	0.11551	0.298449	0.597129

**Table 2:** Turning parameters and measured responses [20].

Turning Parameter			Response	
*v_c_*	*f*	*a_p_*	Ra	TL
6.283	0.01	0.2	1.13	10.511
6.283	0.015	0.5	1.11	5.407
6.283	0.025	1.5	0.629	6.365
6.283	0.02	1	0.616	5.322
12.566	0.01	0.5	0.265	6.359
12.566	0.02	1.5	0.230	3.411
12.566	0.015	0.2	0.849	4.03
12.566	0.025	1	0.378	3.271
18.85	0.01	1	0.056	3.017
18.85	0.015	1.5	0.044	2.567
18.85	0.02	0.2	0.241	2.198
18.85	0.025	0.5	0.221	1.714
25.133	0.01	1.5	0.110	2.237
25.133	0.015	1	0.190	1.878
25.133	0.025	0.2	0.151	1.594
25.133	0.02	0.5	0.049	1.465

**Table 3:** Predicted Ra values based on the regression models in turning.

Actual	LR	PR	SVR	PCR	Quantile	Median	Ridge	Lasso	Elastic Net
0.616	0.7022	0.7805	0.596	0.7276	0.810	0.8191	0.726	0.6433	0.7030
0.849	0.6851	0.6448	0.549	0.6652	0.804	0.8037	0.664	0.506	0.6054
0.378	0.4092	0.2263	0.431	0.4527	0.484	0.4928	0.452	0.4676	0.4797
0.221	0.2786	0.1199	0.255	0.3055	0.338	0.3565	0.306	0.3158	0.3349
0.049	0.0659	0.1684	0.039	0.0709	0.065	0.064	0.073	0.0401	0.0715

**Table 4:** Predicted TL values using the regression models in turning.

Actual	LR	PR	SVR	PCR	Quantile	Median	Ridge	Lasso	Elastic Net
5.322	7.9	9.2765	4.683	6.3661	7.837	5.6544	6.125	6.2169	6.0319
4.03	4.8675	6.2211	5.168	5.8763	7.539	6.069	5.716	5.0261	4.8138
3.271	5.4527	4.704	4.091	3.8531	4.865	3.417	3.881	4.5463	4.7893
1.714	2.8754	2.1036	2.295	2.441	2.364	2.6534	2.639	3.1229	2.5588
1.465	1.8794	3.233	1.885	1.2345	1.988	1.884	1.56	1.8718	2.3406

**Table 5:** Friedman ranks for Ra values in turning.

Test	LR	PR	SVR	PCR	Quantile	Median	Ridge	Lasso	Elastic Net
1	3	7	1	6	8	9	5	2	4
2	7	4	2	6	9	8	5	1	3
3	2	1	3	5	8	9	4	6	7
4	3	1	2	4	8	9	5	6	7
5	5	9	1	6	4	3	8	2	7
Average	4	4.4	1.8	5.4	7.4	7.6	5.4	3.4	5.6

**Table 6:** Friedman aligned ranks for Ra values in turning.

Test	LR	PR	SVR	PCR	Quantile	Median	Ridge	Lasso	Elastic Net
1	12	38	4	23	41	43	22	6	13
2	32	14	5	25	45	44	24	3	7
3	11	1	19	30	37	39	29	33	35
4	15	2	8	27	36	40	28	31	34
5	17	42	9	18	16	26	20	10	21
Average	17.4	19.4	9	24.6	35	38.4	24.6	16.6	22

**Table 7:** *p*-values of Wilcoxon test for Ra in turning.

Model	PR	SVR	PCR	Quantile	Median	Ridge	Lasso	Elastic Net
LR	0.590	0.106	0.178	0.106	0.106	0.178	0.590	0.590
PR	-	1.000	0.590	0.178	0.178	0.590	0.787	0.787
SVR		-	0.059	0.059	0.059	0.059	0.281	0.059
PCR			-	0.106	0.106	0.787	0.281	1.000
Quantile				-	0.281	0.106	0.059	0.281
Median					-	0.106	0.059	0.106
Ridge						-	0.281	1.000
Lasso							-	0.059

**Table 8:** Drilling parameters and responses [33].

Drilling Parameter			Response		
*S*	*f*	*P*	MRR	Ra	O.S
110	0.250	110	75.360	3.665	0.080
110	0.075	110	41.68	2.216	0.135
220	0.075	118	107.65	2.567	0.025
220	0.170	118	160.24	1.725	0.030
220	0.170	118	159.84	1.698	0.025
280	0.170	118	150.72	0.524	0.040
280	0.075	120	118.98	2.973	0.035
220	0.250	118	188.40	1.605	0.030
220	0.170	118	170.40	1.726	0.035
220	0.170	120	160.72	2.332	0.060
280	0.075	110	94.24	2.668	0.130
220	0.170	118	176.76	1.942	0.030
110	0.170	118	83.73	0.948	0.035
280	0.250	120	226.08	1.083	0.035
280	0.250	110	150.72	1.954	0.070
220	0.170	118	165.90	1.856	0.040
110	0.075	120	61.100	2.082	0.025
110	0.250	120	107.65	2.531	0.050
220	0.170	118	164.63	1.958	0.045
220	0.170	110	132.98	2.492	0.143

**Table 9:** Calculated *p*-values of Wilcoxon test for MRR in drilling.

Model	PR	SVR	PCR	Quantile	Median	Ridge	Lasso	Elastic Net
LR	0.295	0.402	0.036 *	0.142	0.142	0.059	0.059	0.059
PR	-	0.529	0.142	0.036^*^	0.142	0.142	0.142	0.142
SVR		-	0.295	0.142	0.142	0.295	0.295	0.295
PCR			-	0.142	0.295	0.295	0.295	0.295
Quantile				-	0.093	0.142	0.142	0.142
Median					-	0.295	0.295	0.295
Ridge						-	0.402	0.529
Lasso							-	0.093

* Significant at 5% significance level (*p*-value ≤ 0.05).

## Data Availability

The data presented in this study are available in the article.
